# Arteriovenous thrombosis, a complication of induction therapy with all‐trans retinoic acid for acute promyelocytic leukemia: A case report

**DOI:** 10.1002/ccr3.7856

**Published:** 2023-09-13

**Authors:** Farhad Tondro Anamag, Negin Hashemi, Zohreh Sanaat, Hengameh Khadivi Heris, Mohammadreza Moslemi

**Affiliations:** ^1^ Hematology and Oncology Research Center Tabriz University of medical sciences Tabriz Iran; ^2^ Department of Pharmacology and Toxicology, Faculty of Pharmacy Tabriz University of medical sciences Tabriz Iran

**Keywords:** acute promyelocytic leukemia, acute abdominal pain, all‐trans retinoic acid, thrombosis

## Abstract

We report a case of arterial and venous thrombosis during induction therapy. This case emphasizes considering some degree of caution for thrombotic events in APL patients which was represented in our case as abdominal pain. Rapid initiation of anticoagulation and preventive measures is suggested for better management of the condition.

## Graphical abstract

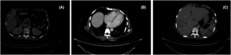

## 1 BACKGROUND

Reciprocal translocation t (15:17) fuses the retinoic acid receptor alpha gene (RARA) with the promyelocytic leukemia gene (PML) and causes myeloid cell lineage to arrest in the promyelocytic phase.^1^ This fusion gives the all‐trans‐retinoic acid (ATRA) the opportunity to play as an efficacious therapy. ATRA, via its capacity to induce myeloid differentiation, eliminates promyelocytic cells and generates remission.^2^

The most dangerous side effect of APL was coagulopathy, which can manifest as either fibrinolysis or disseminated intravascular coagulopathy.^3^ Since the use of ATRA, the bleeding has significantly decreased.^4^ However, thrombosis, a rare complication of APL, not only has not decreased via ATRA therapy but also seems to be a complication of ATRA treatment.^4^, ^5^

We report an uncommon presentation of acute abdominal pain in an APL patient receiving ATRA, complicated by several arterial and venous thromboembolic events.

## 2 CASE PRESENTATION

A 42‐year‐old woman was admitted to the hospital with multiple bruises, in June 2022. She experienced heavy menstrual bleeding and ecchymosis on her limbs. She had grade two fatty liver disease and no thrombophilia in her family history. In the lipid profile, total cholesterol was 197 mg/dL, LDL cholesterol was 144 mg/dL, HDL cholesterol was 56 mg/dL, and triglyceride was 170 mg/dL. On the day of admission, she had numerous ecchymoses on her left groin and forearms with no distinct signs of trauma, infection, or pain. Her complete blood count was pancytopenia. Prothrombin time (PT), international normalized ratio (INR), and activated partial prothrombin time (aPTT) were 12.2 (normal range: 11–13 s), 1 (normal range: 1), and 33 (normal range; 24–40 s), respectively. Physical examination revealed no hepatosplenomegaly or abdominal tenderness. Her body mass index was 34.5 kg/m^2^, and she confirmed a recent weight loss of about 10 kg in 5 months. The patient underwent a bone marrow aspiration and biopsy, which revealed over 50% promyelocytic. We provided leukocytes from the bone marrow, stained them with the following monoclonal antibodies, and analyzed them with flow cytometry. Leukocytes were positive for CD13, CD33, CD45, CD38, CD117, and CD64, weakly positive for CD34 and CD14, and negative for CD2, CD19, CD22, CD3, CD7, CD15, CD10, CD11b, CD41, HLA‐DR, and GlycoA. A reverse transcription polymerase chain reaction (RT‐PCR) study revealed a long isoform of PML‐RARA rearrangement, t (15, 17). The patient, as a low‐risk hyper granular APL, underwent induction therapy with ATRA 90 mg daily (45 mg/m^2^/day), Arsenic trioxide (ATO) 10 mg daily (0.15 mg/kg/day), and Prednisolone 40 mg daily for first 2 weeks (0.5 mg/kg/day) which then tapered to 20 mg daily (0.25 mg/kg/day). She also took low‐dose estrogen‐progestin.

**FIGURE 1:**
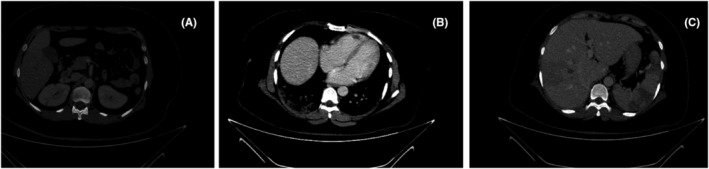
Computed tomography scan of the patient illustrating (A) small hypo‐dense areas in the kidneys, (B) a filling defect in the right ventricle, and (C) multiple wedge‐shaped infarctions in the spleen and hypo‐dense areas in the liver.

The patient developed acute abdominal pain, on the 32nd day of ATRA therapy. The pain was colicky, in the left upper quadrant, radiating to the sternum and left flank, and associated with vomiting. On physical examination, abdomen was soft; Murphy sign was negative, with no tenderness, guarding, costovertebral tenderness, or rebound tenderness. Liver function tests, amylase, lipase, creatinine, and blood urea nitrogen (BUN) were normal. PT, INR, and aPTT were 14.7, 1.4, and 31, respectively. Fibrin degradation products were in the borderline (5–20) range, fibrinogen and D‐dimer levels were 489 mg/dL and 3806 ng/mL, respectively. The patient was negative for antinuclear antibody (ANA), anti‐ds DNA IgG, anti B2 Glycoprotein IgM and IgG, anti Cardiolipin IgM, anti‐Phospholipid IgM and IgG, and Lupus anticoagulant. Likewise, Factor V (Leiden), Protein C, and Protein S were all in normal range. Ultrasonography described no distended common bile duct, kidney stone, hydronephrosis, or ascites. CT scan with intravenous contrast reported thrombosis in the arteries of both inferior lobes of the lungs and right pulmonary vein with infarction foci in comparable lobes of both lungs. Multiple wedge‐shaped hypo‐dense areas in the spleen, suggesting infarction and thrombosis in one branch of the right hepatic vein, were likewise noticeable. Small hypo‐dense areas suggested infarction in the right kidney's superior poles and the middle segment of the left kidney. There was a suspicious filling defect in the right ventricle, which was ruled out by transthoracic echocardiography (Figure 1). As we ruled out disseminated intravascular coagulation, we used enoxaparin to treat these complications. Induction was accomplished, and we achieved complete remission based on repeated bone marrow aspiration. We discharged the patient with no respiratory distress, acute kidney injury, or other organ dysfunction. The patient reached molecular complete remission after four cycles of ATO‐based consolidation therapy and experienced no further thrombotic event in 11 months follow‐up.

## 3 DISCUSSION

The incidence of acute myeloid leukemia increased from 1.35 in 1990 to 1.54 in 2017 per 100,000 population globally.^6^ APL accounts for 5 to 20 percent of acute myeloid leukemia.^7^ While hemorrhage is a known complication, thrombosis is an under‐reported complication of APL, which, with the advent of ATRA, is deriving more attention in clinical practice than before. However, case reports and retrospective studies are the main sources of information on thrombosis in APL.^5^

ATRA stimulates the differentiation of leukemic cells and renders them to lose their capacity to express cancer procoagulant and tissue factor.^8^, ^9^ Although ATRA stimulates cytokine production, it counteracts the TNF‐alpha mediator effect on the down and upregulation of thrombomodulin and tissue factor, respectively, and protects the endothelial wall.^9^, ^10^ These effects reduce the bleeding tendency in APL patients, but thrombosis may be more common in patients treated with ATRA.^5^ The pathogenesis of thrombotic events during induction with ATRA is poorly recognized. It is hypothesized that a rapid decrease of fibrinolytic activity while low‐grade DIC proceeding and high levels of cytokines which overcome the direct effect of ATRA on endothelial cells are possible proposed mechanisms.^11^

The incidence of thrombosis with chemotherapy treatment has been 2%, while studies reported a 16%–19% incidence rate of major thrombosis in patients treated with ATRA.^12^ De Stefano et al. conducted a cohort study, participating in 379 patients with acute leukemia, and reported a cumulative incidence of 8.4% in APL patients, but did not find a significant association between ATRA treatment and thrombosis.^13^ Likewise, in a cohort enrolled with 127 APL patients receiving ATRA, 7.9% of patients developed thrombosis.^14^

In our case, thrombosis occurred during induction with ATRA and complicated arteries and veins in multiple locations, including the lungs, spleen, kidneys, and liver. Although arterial and venous thrombosis are considered distinct entities, a recent meta‐analysis demonstrates common pathways and risk factors.^15^ Rashidi et al. conducted a review article and accumulated 94 cases of thrombosis‐complicating APL patients. He reported that 43.6% of patients developed thrombosis during induction therapy and deep vein thrombosis, pulmonary thromboembolisms, myocardial infarctions, and cerebrovascular accidents made up over three‐quarters of cases. Furthermore, only six cases developed thrombosis in multiple body sites.^12^

Searching the literature, there are controversial evidence about the risk factors of thrombosis in APL patients. Our case was obese and meta‐analysis has revealed that obesity and high BMI are associated with an increased risk of differentiation syndrome, shorter overall survival, and adverse clinical outcomes.^16^ Moreover, it has been established that obesity induces an inflammatory state and stimulates the production of cytokines like TNF‐alpha which may play a role in thrombosis formation in APL patients.^17^ Our patient took Levonorgestrel 0.15 mg and Ethinyl Estradiol 0.03 mg daily for vaginal bleeding. Although there is no evidence concerning the increased risk of thrombosis in APL patients receiving ATRA, this medication, especially at high doses, may be a precipitating factor of thrombosis.^18^ Breccia et al. proposed high leukocyte count, bcr3 isoform of the fusion protein, and expression of FLT3‐ITD, CD2, and CD15, but not differentiation syndrome as risk factors.^19^ Other studies revealed variant subtypes of APL, differentiation syndrome, low fibrinogen level, antifibrinolytic therapy, and tranexamic acid as potential risk factors, and ruled out FLT3‐ITD, CD2, and CD15.^20^ None of the potential risk factors except tranexamic acid were present in our case. It has been postulated that tranexamic acid and other antifibrinolytic agents with ATRA could further exacerbate the procoagulant state.^21^

## 4 CONCLUSION

Thrombotic events are rare in APL patients. However, unexplained acute abdominal pain should be suspected of thromboembolic events, especially during induction therapy. Anticoagulants like enoxaparin seem to be a safe and effective treatment in these patients, and predisposing factors like BMI should probably be considered at the beginning of therapy to make more convenient clinical decisions and supportive therapy.

## AUTHOR CONTRIBUTIONS

**Farhad Tondro Anamag:** Conceptualization; data curation; writing – original draft. **Negin Hashemi:** Investigation; writing – original draft. **Zohreh Sanaat:** Data curation; writing – review and editing. **Hengameh Khadivi Heris:** Project administration; supervision; writing – review and editing. **Mohammadreza Moslemi:** Writing – review and editing.

## FUNDING INFORMATION

None declared.

## CONFLICT OF INTEREST STATEMENT

None declared.

## PATIENT CONSENT

Written informed consent was obtained from the patient prior to submission of the draft.

## Data Availability

All data supporting the study are presented within the article.
